# Theoretical Predictions of Lactate and Hydrogen Ion Distributions in Tumours

**DOI:** 10.1371/journal.pone.0072020

**Published:** 2013-08-21

**Authors:** Maymona Al-Husari, Steven D. Webb

**Affiliations:** 1 Department of Mathematics and Statistics, University of Strathclyde, Glasgow, United Kingdom; 2 MRC Centre for Drug Safety Science, Department of Molecular and Clinical Pharmacology, Institute of Translational Medicine, The University of Liverpool, Liverpool, United Kingdom; University of Leeds, United Kingdom

## Abstract

High levels of lactate and H^+^-ions play an important role in the invasive and metastatic cascade of some tumours. We develop a mathematical model of cellular pH regulation focusing on the activity of the Na^+^/H^+^ exchanger (NHE) and the lactate/H^+^ symporter (MCT) to investigate the spatial correlations of extracellular lactate and H^+^-ions. We highlight a crucial role for blood vessel perfusion rates in determining the spatial correlation between these two cations. We also predict critical roles for blood lactate, the activity of the MCTs and NHEs on the direction of the cellular pH gradient in the tumour. We also incorporate experimentally determined heterogeneous distributions of the NHE and MCT transporters. We show that this can give rise to a higher intracellular pH and a lower intracellular lactate but does not affect the direction of the reversed cellular pH gradient or redistribution of protons away from the glycolytic source. On the other hand, including intercellular gap junction communication in our model can give rise to a reversed cellular pH gradient and can influence the levels of pH.

## Introduction

The microenvironment of tumours has been shown to trigger various signals which promote invasion [1], [2] and reduce tumour response to therapies [3], [4]. An altered pH homoeostasis is increasingly becoming a distinct feature of some cancer cells [5]. While the intracellular pH (pH_i_) in normal differentiated cells is generally ∼7.2 and is less alkaline than the extracellular pH (pH*_e_*∼7.4) [6], the intracellular pH of some malignant tumour cells can be greater than 7.4 and is found to be more alkaline than the extracellular pH (pH*_e_*∼6.5–7.1) [7], [8]. This gives rise to a reversed cellular pH gradient (pH*_i_*>pH*_e_*) – also known as a negative cellular pH gradient (pH*_e_*–pH*_i_*<0) – in these tumours which is thought to confer a survival advantage to the tumour over normal tissue [9], [10]. An acidic pH*_e_* has been shown to enhance the invasive behaviour of tumour cells [1], [11] and render them resistant to some chemotherapeutics [12], [13]. On the other hand, an elevated pH*_i_* has shown to have permissive effects on proliferation [14], [15], the evasion of apoptosis [16], [17] and is necessary for directed cell migration [18].

More than 80 years ago, Warburg [19] observed that tumour cells exhibit an altered metabolism, marked by increased glucose uptake and elevated glycolysis. In the absence of oxygen, pyruvate is converted into two molecules of lactic acid which dissociates rapidly into lactate and H^+^ ions [20]. Warburg’s pioneering work also showed that even in the presence of an ample supply of oxygen, tumour cells still undergo anaerobic glycolysis [19]. This type of energy metabolism is inefficient compared to aerobic metabolism and, for a vastly growing tumour to maintain sufficient production of ATP, the tumour cells must up-regulate their glycolytic pathway. As a result, more lactic acid is produced and the tumour can become very acidic [19]. In fact, tumours were initially thought to have an acidic intracellular pH (pH*_i_*). But, the invention of non-invasive measurements of pH*_i_* by magnetic resonance spectroscopy (MRS) has shown that tumour pH*_i_* can actually be alkaline [21].

The metabolically produced hydrogen ions must be extruded to ensure a physiological pH*_i_* and maintain cell viability. This is because many cellular processes such as those associated with metabolism [22], the cell cycle [23], [24] and cell proliferation [25], [26] are all pH sensitive. Furthermore, most mammalian cells will not proliferate at a pH less than 6.6 [25]. Cells, therefore, have evolved several short and long term mechanisms to maintain their pH*_i_* within the normal physiological range (pH 7.2–7.4). Short term homoeostasis, for example, involves a rapid defence mechanism that minimises changes in pH as a result of acid or alkali load [25]. This includes physicochemical buffering, H^+^–consuming metabolic buffering and organelle sequestration or release of hydrogen ions [25].

In addition, cells employ another strategy to maintain their pH through several membrane-based transport systems. The universal membrane protein, Na^+^/H^+^ antiporter exports one H^+^ ion outside the cell in return of one Na^+^ ion [27]. This antiporter plays an essential physiological role in the regulation of cytoplasmic pH, and a change in its activity can have a drastic effect on cell metabolism and viability [27]. The Na^+^/H^+^ antiporter is freely reversible depending on both the cellular Na^+^ and H^+^ gradients. However, most mammalian cells maintain an inward cellular Na^+^ gradient which stimulates H^+^ ions efflux. This process is tightly mediated by pH and the antiporter’s activity changes by more than three orders of magnitude between pH 7 and 8 (recall that pH = –log[H^+^]) and is totally down-regulated below pH 6.5 [27].

A key pH transmembrane exchanger is the lactate/H^+^ symporter (also known as MCT–Monocarboxylate Transporter) [28]. This symporter works by transporting lactate and hydrogen ions together in the same direction. Depending on the cellular gradient of each ion, this process is freely reversible with equilibrium being attained when [lactate*_i_*]/[lactate*_e_*] =  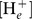/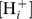. There is a growing evidence suggesting that elevated tissue lactate levels are associated with a high risk of metastasis [28], [29] and a reduced response to radiotherapy [30]. Moreover, reports by Cardone *et al.* [31] claim that the lactate/H^+^ symporter and the Na^+^/H^+^ antiporter cause tumour acidity which in turn stimulates metastasis.

The contributions of mathematical modelling to the understanding of tumour growth and development dates back at least 60 years. Models mainly explore particular aspects of tumour growth and dynamics such as immunotherapy (e.g. see [32]), angiogenesis (e.g. see [33]) and invasion (e.g. see [34], [35]). However, there are only relatively few mathematical models that consider tumour acidity. Amongst these are the work of [35]–[39]. Gatenby & Gawlinski [36] derive an acid-mediated tumour invasion model which provides a simple mechanism linking altered glucose metabolism with the ability of tumour cells to form invasive cancers. The modelling of Webb *et al.* [38], [39] includes descriptions of intracellular and extracellular pH and their effects on invasion. However, in this work the various cell-membrane transporters are represented in a simplified fashion. Moreover, they do not include lactate as a variable, but instead include the lactate/H^+^ symporter as a function depending wholly on extracellular H^+^ and the degree of functioning vasculature. The role of sequestration of H^+^-ions into lysosomes is also considered in [39]. The modelling of Neville *et al.* [37] considers the evolution of intracellular and extracellular glucose as well as hydrogen ions.

Recently, we developed an ordinary differential equation (ODE) model for pH regulation that explicitly focuses on the interplay between H^+^-ions and lactate [40]. Analysis of this model showed that a reversed cellular pH gradient is attainable under aerobic conditions when the MCT activity is increased and other sources of H^+^-ions decreased–but we find the pH conditions predicted are too alkaline to be viable and therefore is unrealistic. To increase the biological realism of this earlier work we extend the model in this study to include spatial heterogeneity of lactate and H^+^-ions. In so doing, we also examine the findings of Provent *et al.* [41] that predict, in some cases, the spatial concentrations of extracellular lactate and extracellular hydrogen ions are often uncorrelated. We begin this study by investigating the conditions under which this phenomenon is observed. It is suggested that this is because protons, which are exported outside cells along with lactate in hypoxic regions, re-enter the cells indirectly via the 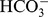/Cl^–^ exchanger or simply leak back into the cell and then are transported cell-to-cell via gap junctions to make protons available for the NHE exchanger [41]. Another study by Grillon *et al.* [42] reports that the distribution of NHEs and MCTs in rat brain gliomas are heterogeneous–the relative intensity of NHE1 (isoform 1) peaks at an average distance of 0.33±0.027 mm from the edge of the tumour and expression of the MCT1 (which can transport lactate and H^+^ either out of or into cells [43]) peaks further into the glioma (1.05±0.14 mm from the edge of the tumour). We also incorporate these findings into the model and examine their effect on the cellular pH gradient. We then finally investigate the effect of H^+^-ion intercellular gap junctions on the cellular pH gradient reversal and the spatial distribution of extracellular lactate and H^+^-ions.

## Methods

**Figure 1:**
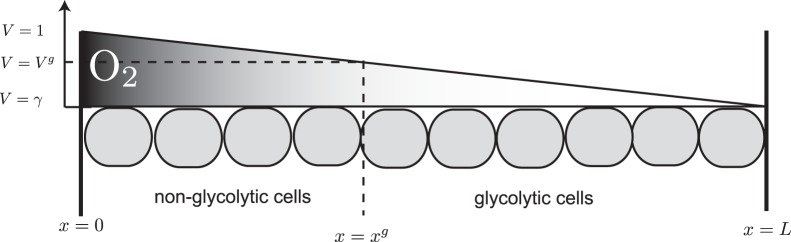
A schematic representation of the gradient of oxygen and the corresponding distribution of glycolytic (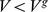) and non-glycolytic cells (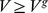) in the model (1)–(4). 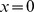
 denotes the location of the blood vessel.

We model a 2-D slice through a three-dimensional tumour mass, but we average the dependent variables in the plane perpendicular to the edge of the tumour to reduce the problem to one that is one-dimensional. Our modelling domain is between the blood vessel and 2 mm into the tumour mass. We restrict ourselves to this because we have corresponding experimental data for this size of section. We assume that the tumour extends beyond 2 mm and we assign appropriate perfusions of the chemical constituents into this extended region. The cells have two compartments–intracellular and extracellular–and we focus on the regulation of lactate and H^+^ between these two compartments. The extracellular space in our model represents the small interstitial spaces between the tumour cells. We assume that the volumes of these intra- and extracellular compartments are comparable and we take them to be equal in our analysis. Our model considers the spatial and temporal evolution of H^+^ which we denote by 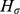, 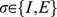 where 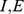 denotes intracellular and extracellular concentrations, and lactate (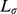) where 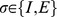. We define the cellular pH gradient to be pH
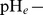
pH*_i_* and the cellular lactate gradient to be 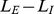. For simplicity, we assume a one-dimensional Cartesian geometry, namely 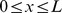, where 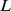 denotes the distance away from the blood vessel which is located at 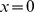. Rather than including the complexity of an additional equation for oxygen, we simply assume a linear decreasing concentration of oxygen (denoted by 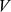) from the blood vessel located at 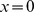, namely 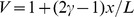, where 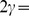 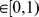 (see Fig. 1 for a schematic). Note that similar to [40] we have rescaled oxygen to be one at the blood vessel. With appropriate choices of 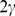, we can either simulate a tumour which is completely well-oxygenated (e.g. if 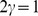) or a tumour that is hypoxic for 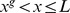 and aerobic for 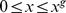, where 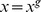 is the point beyond which glycolysis prevails.

Our model has the form
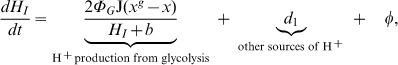
(1)

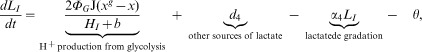
(2)

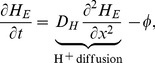
(3)

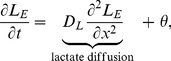
(4)where,



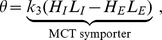


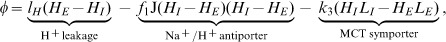
and 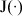 is a Heaviside function, which is a binary function, being one if the dependent variable is positive and zero otherwise.

We first clarify each of the model expressions in turn. Note that there are more detailed models for cellular ion transport based on the Goldman-Hodgkin-Katz equations – which play an important role in models for cellular electrical activity. However, we adopt a simple phenomenological approach based on experimental observations of transport rates as functions of ion concentration. For instance, the Na^+^/H^+^ transporter term is taken to be linear and uni-directional based on experimental observations by Boyer & Tannock [44]. This type of modelling approach has been successfully adopted previously by, for example, Webb *et al.* [38], [39], Al-Husari & Webb [40], Neville [37], Vinnakota & Beard [45], Aubert & Costalat [46].

**1.** 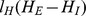


This term describes the rate at which H^+^ ions enter the cell due to the internally negative potential of the cell membrane. It is assumed to be directly proportional to the difference in the hydrogen ion concentration across the cell membrane. The permeability of the cell membrane to H^+^ ions is approximately 10^–14^ m/s [47]. Dividing this by the typical width of the bilayer (∼10 nm) [47] gives an estimation for 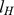 as 10^−6^ s^−1^.

**2.** 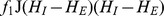


This term models the rate at which H^+^ ions are exported outside the cell via the Na^+^/H^+^ exchanger (NHE for short) and we assume that the rate of H^+^ efflux is directly proportional to the cellular H^+^ gradient across the cell membrane, i.e. 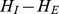. This is based on experiments carried out by Boyer & Tannock [44] that report that the rate of H^+^ efflux is linearly proportional to the transmembrane H^+^ gradient (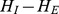) in MGHU1 human bladder carcinoma cells and unidirectional for the pH ranges considered. The function J is used to prevent any H^+^ influx which is typically not observed via this transporter. The constant 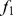 is a parameter which denotes the rate of H^+^ flux, and carries the units of s^−1^.

**3.** 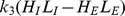


This term represents the rate at which hydrogen ions are extruded along with lactate ions. These ions are transported via a Monocarboxylate Transporter (MCT) located at the plasma membrane. A study by McDermott *et al.* [48] showed that lactate transport is saturable with respect to increasing concentrations of lactate and hydrogen ions, but for simplicity we assume that this transporter is acting in the linear regime–a full derivation of this term is given in [40]. The constant 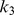 (mol^−1^/l^−1^/s) describes the rate at which hydrogen ions and lactate are exported or imported.

**4.** 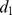


This term implicitly accounts for sources of H^+^ ions in the cell other than via glycolysis. For example, this could include the catalysed hydration of CO_2_ into H^+^ and 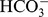 by Carbonic Anhydrase [49]. This buffering parameter is a leading order representation of a process that is short term, in relation to ion pumps which act over the long term to reduce acidosis, and hence we assume it to be quasi-steady and taken to be constant.

**5.** 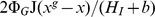


This term models the net production of H^+^ ions via the process of glycolysis. Glycolysis is a metabolic pathway involving a complex chain of chemical reactions that produces energy rich molecules (ATP) [47]. Studies by Kaminkas [50] showed that glucose transport and consumption in cultured Ehrlich ascites-tumour cells are pH dependent. Decreasing pH*_i_* is found to decrease the rate of glucose consumption [25], [51]. In particular, the key glycolytic enzyme phosphofructokinase is found to be critically pH sensitive [20]. This dependency of intracellular H^+^ is also included by Neville [37] in her model for tumour glycolysis. In our model, we assume a threshold degree of vasculature (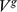), above which a cell will undergo aerobic metabolism, and below which anaerobic glycolysis will prevail. We define how vasculature is included in the model below. In the presence of an oxygen supply (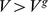), there is no net production of H^+^ ions as aerobic metabolism is shown not to produce any net H^+^-ions [52]. However, in low oxygen concentrations (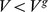), two H^+^-ions are produced from the dissociation of lactic acid [52]. We assume glucose to be plentiful, which is reasonable given the observed large diffusion distance of glucose [53]. The constant 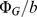 represents the maximal rate of glycolysis. We use the results of [51] for EMT6/R0 mouse mammary tumour cells to estimate 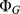 and 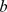. In this study, it is noted that glucose is consumed at a rate of 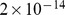 g/cell/s at a pH of 7.2. One mol of glucose has a relative molecular mass of 180 g and one cell has a volume of roughly 10^−15^ m^3^ [54]. This corresponds to a glucose consumption rate of 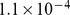 mol/m^3^/s. If we choose 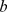 = 10^−7^ mol/l then 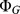 = 10^−14^ (mol/l)^2^/s. We assume that 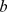 does not change between normal and tumour cells. However, tumours are known to have a higher glycolytic rate than normal cells and we represent this excess by an increase in 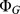.

**6.** 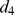


Even under aerobic conditions, there is evidence to suggest that there is some degree of lactate production [55]. Lactate is known to be only produced via the breakdown of pyruvate which is made from either glucose or some amino acids [56]. Therefore, since our model assumes no production of lactate from glucose under aerobic conditions, 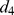 may still account for a minor production from glucose. On the other hand, under anaerobic conditions, 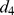 may account for lactate production from some amino acids. In non-stressed or non-shocked animals, significant lactate is produced to maintain a concentration of 0.7 mM [57]. It has been estimated [57] that lactate is produced in the resting human at the following rates (mM/h/kg): skeletal mass, 3.13; brain, 0.14; red cell mass, 0.18; and 0.11 for blood elements, renal medulla, intestinal mucosa and skin. Total lactate production in a 70-kg male is approximately 1,300 mM/day [57].

**7.** 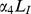


This term implicitly describes the rate at which lactate is converted back to pyruvate. That is, if we assume a linear conversion from pyruvate to acetyl-coA and steady state efflux conditions then one can estimate a linear relationship between pyruvate and lactate concentrations and then obtain a linear loss term for lactate, namely 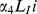. A similar approach has been adopted in Bertuzzi *et al.* [58]. We currently have no available data to approximate this value and so we vary it in our analysis.

The current model differs from our recent work in [40] via the added diffusion terms of extracellular H^+^-ions and lactate, with diffusion coefficients 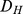 and 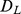 respectively. Also, here the boundary conditions at 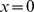 replace the terms, 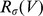, 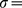
1 and 2, used in [40] for the vascular removal of extracellular H^+^-ions and lactate, respectively. We assume that glucose supply is plentiful 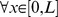. We impose boundary conditions to represent a tumour with a well-perfused blood vessel (on the left side of the tissue, 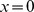) which supplies the tumour with oxygen and removes H^+^Z-ions and lactate. That is.
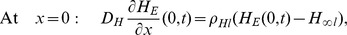


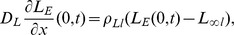
where 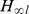 and 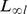 are, respectively, the concentrations of hydrogen ions and lactate inside the blood vessel at 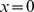. Their rate of leakage into or out of the blood vessel at 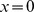 is regulated by the parameters 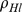 and 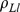 respectively. A similar notation is used for lactate at the right hand side boundary condition, but with 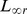 denoting estimated tissue lactate levels:



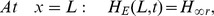


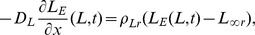



Based on an experimental observation, we take a fixed boundary condition at 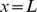 for the extracellular H^+^ since findings show that at 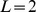 mm, the extracellular pH is known to be around 6.5 (Personal Communication with Jonathan Coles, Institute of Photonics, University of Strathclyde). Our rationale is that beyond 2 mm the environment is too hypoxic to allow sufficient cell metabolism and thus we expect the net production of lactate and H^+^ to be low, therefore facilitating the inflow of these constituents from the high producing 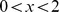
mm tumour region. We cannot find appropriate values for the tumour tissue lactate at or beyond 2 mm and so we do not adopt the same boundary condition at *x* = 2 mm for lactate as we do for pH*_e_*. However, we take it to be of the same order as that of normal blood lactate but, ultimately, we find that the solutions are not very sensitive to the particular value chosen.

We denote the initial values by their normal concentration in the tissue, namely, 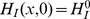 mol/l, 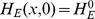 mol/l, 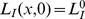 mol/l, 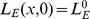 mol/l.

### Non-dimensionlisation

To facilitate the numerical study of the model, we rescale the system using the following rescalings, where the tilde represents the rescaled space variable:
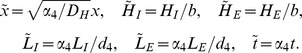



The dimensionless equations then read
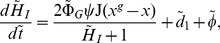
(5)

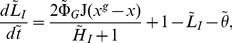
(6)

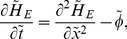
(7)

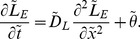
(8)


See Table 1 for a list of how the dimensionless parameters are calculated and their estimated values.

**Table 1:** Dimensionless parameter estimates used in the one-dimensional spatial model in (5)–(8).

Description	Symbol	Definition	Value	Ref.
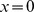 Vessel permeability to H^+^ at	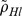	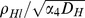	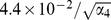	Estimate
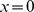 Vessel permeability to lactate at	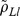	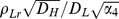	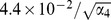	[61]
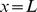 Tissue permeability to lactate at	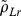	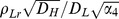	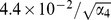	Estimate
Normal H^+^ concentration in blood	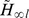	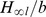	0.35–0.45	[12]
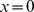 Lactate concentration in blood at	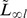	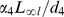	0.35–0.71	[63]
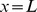 Lactate concentration in the tissue at	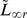	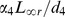	1.42	[62]
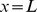 H^+^-ions concentration in the tissue at	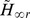	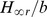	3.16	PC
Diffusion co-efficient fraction	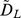	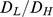	0.81	[59], [60]
Tissue size	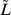	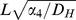	0.02	PC
Rate of H-leakage inside the cell	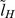	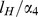	1.7174×10^−2^	[47]
Rate of NHE activity	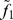	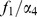	1.7174×10^4^	Estimate
Rate of MCT activity	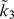	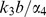	5.4316	Estimate
Background production of intracellular H^+^	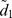	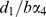	7.9996×10^3^	Estimate
Rate of glycolysis	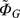	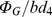	0.2823	[51]
Scaling factor	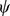	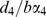	1.4×10^4^	Estimate
Initial intracellular H^+^ concentration	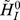	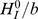	0.63	[6]
Initial extracellular H^+^ concentration	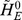	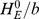	0.63	[6]
Initial intracellular lactate concentration	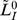	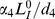	1	[63]
Initial extracellular lactate concentration	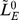	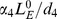	1	[63]

PC = personal communications with Jonathan Coles, Institute of Photonics, University of Strathclyde.

## Results

### Spatial Discretisation and Numerical Scheme

We divide the spatial domain into 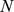 uniformly spaced points with grid size, 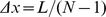. This allows the problem to be solved by the method of lines and gears using MATLAB’s built-in ODE solver (ode15s) with four ODEs in time (for 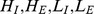) at each space point. The grid function 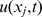, 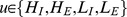, denotes an approximation of 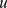 at 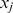, where 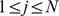. For the diffusion terms of 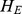 and 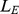, we use a simple finite difference approximation using a second order central difference.

### Parameter Estimates

From the literature, we are able to estimate some of the model parameters. We take the diffusion co-efficient of H^+^ ions (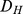) to be 1.08×10^−5^ cm^2^/s [59] and that of lactate (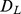) to be 8.8×10^−6^ cm^2^/s [60]. We assume that cells near the blood vessel are well-oxygenated and that extracellular lactate and H^+^-ions leak in or out of the blood vessel at a much higher rate than extracellular lactate does into the tissue at 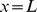 due to the leakiness of the tumour vasculature. The vessel permeability to lactic acid (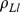) is taken to be 1.19×10^−4^ cm/s [61], for which the non-dimensional equivalence is 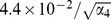. With a lack of available data, we also assume that H^+^ ions have the same vessel permeability as lactate. Also, because of the lack of available data on the rate of lactate decay (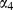), we vary this parameter in our study and note the effect on the overall model behaviour.

In general, normal blood lactate in unstressed patients is between 0.5–1.0×10^−3^ mol/l, but for patients with critical illnesses, concentrations of more than 2.0×10^−3^ mol/l are sometimes found [62]. With this in mind, we estimate lactate levels inside the blood vessel at 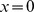, 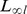, to be 0.5–1.0×10^−3^ mol/l [63], and lactate 2 mm away from the blood vessel, 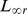, to be 2.0×10^−3^ mol/l [63]. From our parameter estimates in [40] we find that 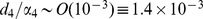 mol/l, which gives 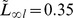
–0.71 and 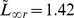 (in dimensionless form). The normal blood H^+^ concentration in unstressed patients is found to be in the range of 3.55–4.5×10^−8^ mol/l [64] and we take this value to represent 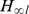.

Almost fifty years following Warburg’s pioneering work on tumour metabolism [19], extensive studies have concluded that glucose is a main energy source for malignant tumours [65], [66] and that 60% of cancer cells are glycolytic [67]. We therefore partition our tumour section so that 60% of the cells undergo anaerobic glycolysis and the remaining 40% do not. For details on the derivation of the remaining parameter estimates, see [40]. A summary of the non-dimensional parameter values used in the model is presented in Table 1 (we will refer to this as the base set of parameters). We simulate the model with appropriate non-dimensional initial conditions that represent normal tissue levels: 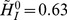, 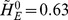, 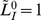 and 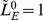. We will work with the non-dimensional model hereafter but drop the tildes for notational convenience.

### Spatial Distribution of Extracellular Lactate and Hydrogen Ions

**Figure 2:**
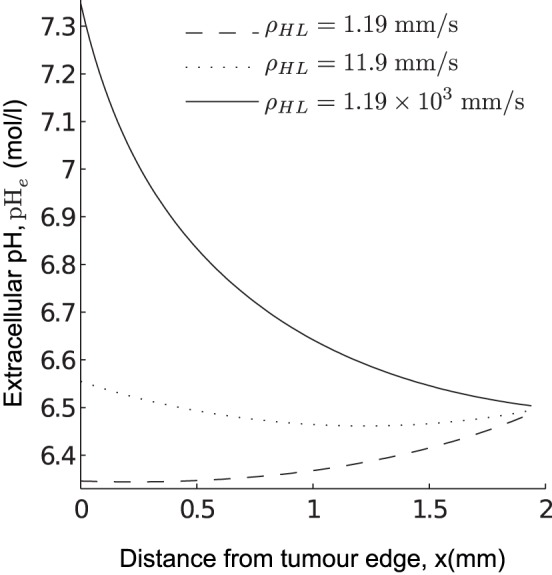
Effect of varying the rate of H^+^ leakage into the blood vessel (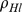) on pH*_e_*. We show how pH*_e_* near the blood vessel varies as the rate of H^+^ leakage into the blood vessel varies. Parameter values are as in Table 1 and with 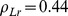, 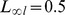, 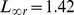, 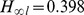. The prescribed model is simulated until the steady state solution is reached, starting from the initial conditions: 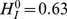, 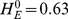, 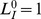 and 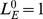. We determine whether the steady state solution is reached by considering the solution at time step, 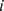, and time step, 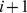. If the absolute difference in solution is less than some 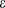 (we choose to be 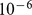), then the model stops running at the 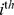 step. With this value of 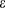, the metabolites reach a steady state at 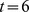.

Given that there is some uncertainty in the values that we should take for the vessel permeabilities (for example, 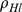) – mostly stemming from the uncertainty of the value of 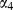 (i.e. recall that 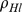 (dimensionless) 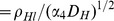 – we first vary the dimensionless value of 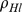 in our analysis. In particular, we are interested to find if this parameter has any effect on whether the extracellular pH is less acidic when extracellular lactate is high. Fig. 2 shows how the spatial profile of extracellular pH at steady state is qualitatively reversed (i.e. from that with an increasing pH*_e_* profile against space to that of a decreasing pH*_e_* profile against space) as 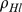 increases. Note that as 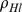 increases, pH*_e_* slowly increases near the blood vessel and attains a minimum parabolic profile which gradually changes into a monotonically decreasing function as 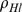 further increases. We find that the spatial profile of extracellular lactate, however, remains qualitatively unchanged as 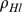 increases (data not shown).

**Figure 3:**
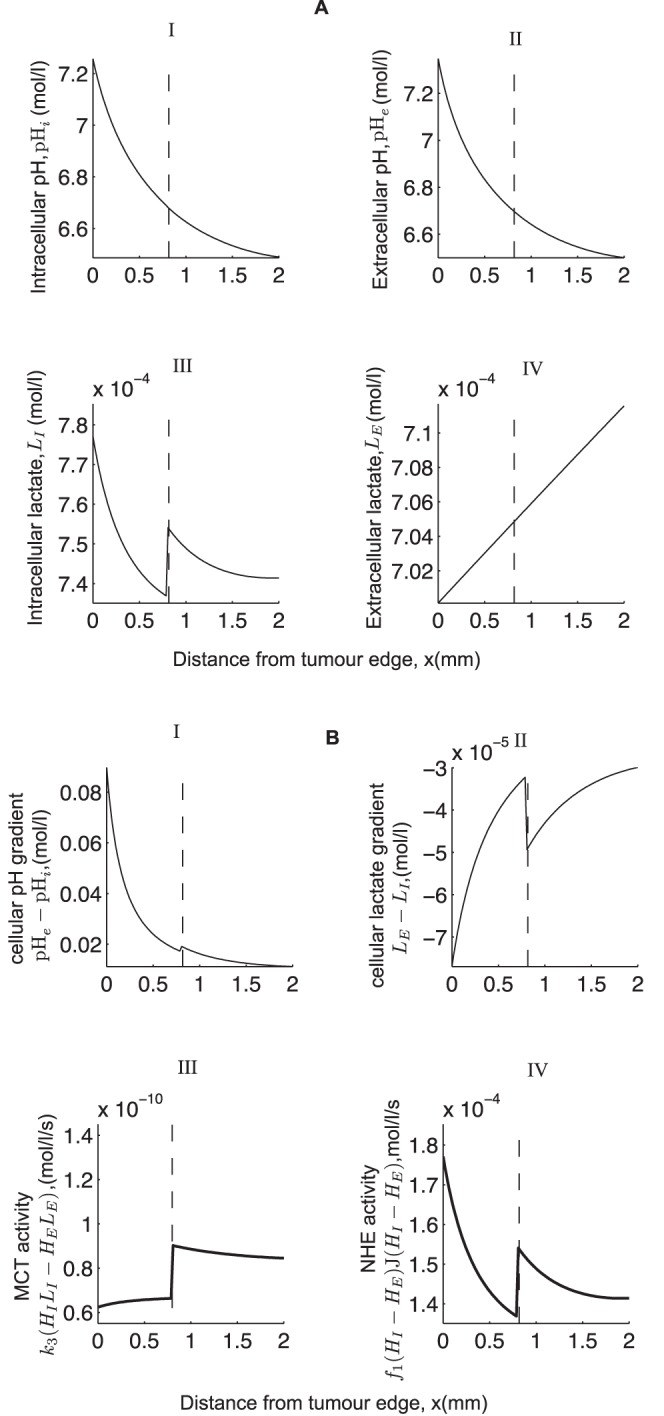
Numerical solution of the system (5)–(8) showing a correlation between pH*_e_* and 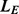. The vertical dashed lines denote 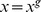, where here 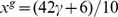, with 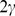 chosen to be 0.1 throughout the simulations in this study. (A) A spatial correlation exists between extracellular lactate levels and pH*_e_* (e.g. compare (A) II and (A) IV). (B) I shows the pH gradient, (B) II the lactate gradient, (B) III the MCT activity and (B) IV the NHE activity. Parameter values are as in Table 1 with 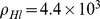, 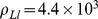, 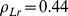, 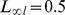, 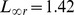, 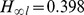. The prescribed model is simulated until the steady state solution is reached, starting from the initial conditions: 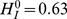, 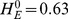, 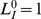 and 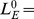
1. We determine whether the steady state solution has been reached as in Figure 2.

We show a typical simulation with large 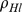 and 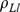 ( = O(10^3^)) in Fig. 3. Note the “jump” in the intracellular concentration profiles and the activity of the membrane-based transporters observed at 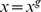. This is due to the switch from aerobic metabolism in the 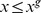 region to anaerobic metabolism in the 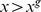 region where the oxygen levels are low. The extracellular lactate and pH display no significant observable “jump” in their profile due to the smoothing effect of the extracellular diffusion. The key solution features are as follows: intracellular pH and extracellular pH are lower and extracellular lactate is higher in the region further away from the blood vessel (i.e. in the 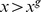 region) than that in the aerobic region close to the blood vessel. However, intracellular lactate levels increase in the anaerobic region but quickly drop to levels below that found close to the blood vessel. This could be because the NHE activity near the blood vessel is high due to the larger H^+^-ion transmembrane gradient as a result of an increased removal of extracellular H^+^-ions into the blood vessel. Consequently, the MCT activity near the blood vessel becomes low because there are less intracellular H^+^-ions available for extrusion and as a result intracellular lactate levels near the blood vessel become high and decrease as the activity of the NHE decreases (away from the blood vessel).

**Figure 4:**
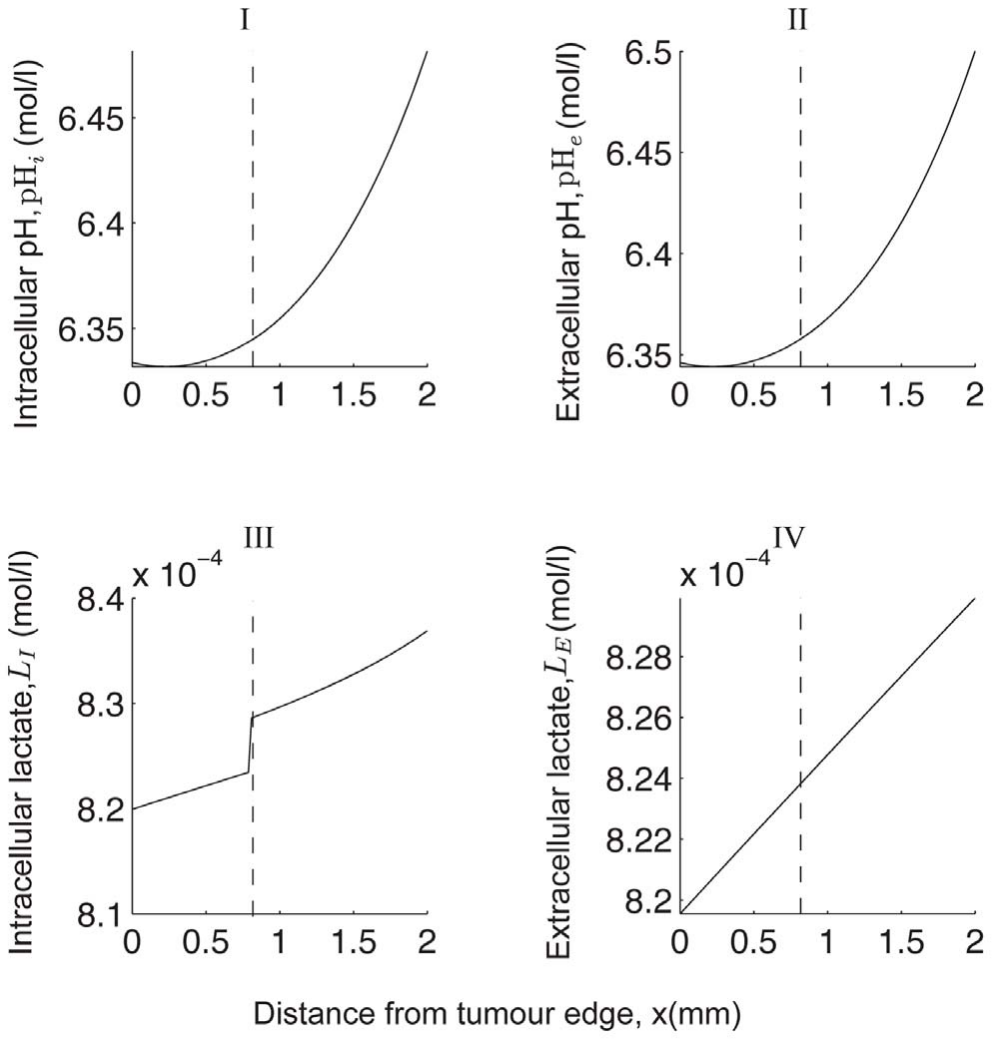
Numerical solution to equations (5)–(8) showing a lack of spatial correlation between extracellular H^+^ and 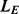. This is obtained using parameter values as in Fig. 3 but with a reduced rate of leakage of H^+^-ions and lactate into the blood stream (i.e. 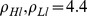), compare II with IV. The model is simulated until the steady state solution is reached, starting from the initial conditions: 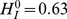, 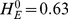, 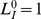 and 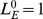. We determine whether the steady state solution is reached the same way as stated in the caption for Fig. 2. Note that the spatial profiles for the cellular pH gradients, MCT and NHE activities are qualitatively similar to the plots for pH*_i_*, L*_I_* and L*_E_*, respectively, and so we omit them for brevity.

Note that, a lack of spatial correlation between extracellular acidity (H^+^-ions, recall that 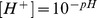) and extracellular lactate is predicted by the model, as shown in Fig. 4. This is in line with the findings of Provent *et al.* [41] which showed that the glucose-induced increase in extracellular lactate showed no associated decrease in extracellular pH. However, they suggest that the re-distribution of extracellular H^+^-ions at sites remote from anaerobic lactate production is primarily due to the leakage of H^+^-ions intracellularly and their subsequent transfer by gap junctions to make them available for extrusion by the NHEs. In contrast, our model predictions suggest that this same result can be observed in the absence of gap junctions, and instead with a reduced permeability of the blood vessels to H^+^-ions and lactate. In a biological sense, we may interpret our results by suggesting that less-efficient blood vessels, which are indeed frequently found in tumours, can give rise to a contrasting spatial distribution of extracellular pH and lactate.

### Effect of Parameter Variations on the Heterogeneity of the Cellular pH Gradient

In this section, we study the extent of the reversed cellular pH gradient in the spatial context and show that, in some cases, the extracellular environment is more acidic than the intracellular for all the cells in the domain or only for non-glycolytic cells or in other cases, no negative cellular pH gradients are found in any region of the tissue section considered. Our parameter sensitivity analysis of the well-mixed ODE model in [40] shows that the activity of the MCTs (represented by 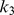), NHEs (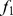) and other sources of intracellular H^+^-ions (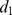) play a crucial role in the model behaviour. We now explore whether these parameters are similarly important within this new spatial framework.

#### Varying the concentration of extracellular lactate in the blood vessel versus that in the tissue

**Figure 5:**
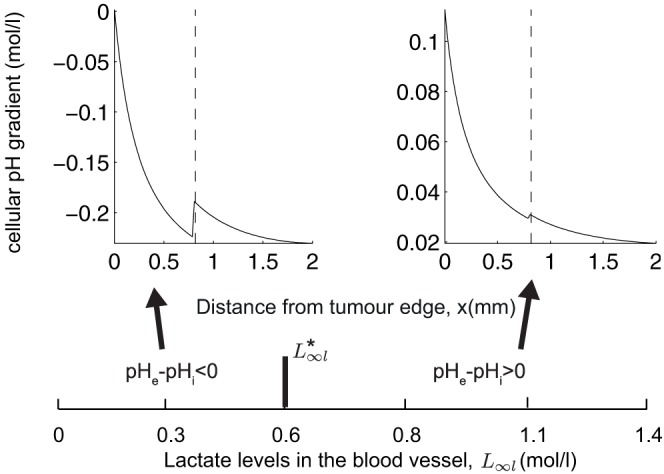
Effect of varying blood lactate levels (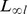) on the cellular pH gradient pH*_e_*-pH*_i_*. For 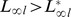 all the cells exhibit a negative cellular pH gradient; for 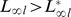 all cells have a positive cellular pH gradient. Shown above the horizontal panel are typical spatial profiles of the cellular pH gradient in the two cases 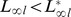 and 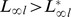. For each set of chosen parameters, the model is simulated until the steady state solution is reached, starting from the initial conditions: 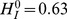, 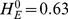, 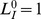 and 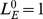. We determine whether the steady state solution is reached the same way as stated in the caption to Fig. 2. 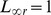 in each subplot and remaining parameters are the same as in the caption to Fig. 3.

Recall that 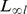 and 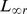 denote the concentrations of extracellular lactate in the blood and tissue respectively. Exploring simulation solutions of our model, we find that simply taking lower values of 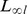 causes a reversed cellular pH gradient across all the cells in the tissue section, as illustrated in Fig. 5. For example, with 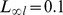, the cellular pH gradient is reversed throughout the entire spatial domain considered. This is because, if we take 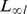 to be very small, 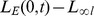 in the boundary term at 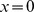 is likely to be positive and large, which means that extracellular lactate will leak into the blood stream at a high rate and hence the levels of extracellular lactate throughout the tissue section will be low. This will then facilitate the activity of the MCT (which functions according to the cellular lactate and H^+^ gradient) and consequently will result in a reversed cellular pH gradient as H^+^-ions are exported outside the cells along with lactate. We note that in this case, the observed reversed cellular pH gradients occur at realistic values (pH*_e_* 6.5–7.4 for the parameters used in Fig. 5) in contrast to our modelling predictions in the well-mixed version of this model, see [40]. As 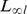 increases beyond a threshold value (which we donote by 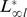), the perfusion rate of lactate into the blood decreases, tissue lactate then increases and the MCT activity subsequently decreases. All cells then exhibit a positive cellular pH gradient. Our model therefore predicts that low levels of lactate found in the blood stream may indirectly cause a reversed cellular pH gradient in conjunction with an up-regulated tumour activity of the MCT.

#### Varying the activity of MCTs and other sources of intracellular H^+^-ions

**Figure 6:**
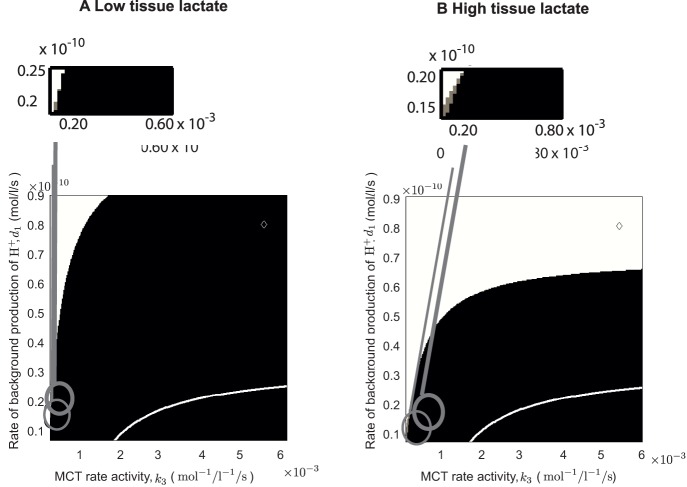
Effect of varying the MCT rate activity (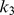) and the background production rate of H^+^ (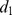) on the cellular pH gradient in the cases of high and low tissue lactate (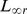). In (A) low tissue lactate (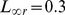) and (B) high tissue lactate (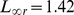). The remaining parameters are the same as in the caption to Fig. 3. For parameters in the black shaded region all cells have a negative pH gradient (i.e. 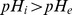). In the white regions, 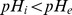. We also superimpose the results from our well-mixed ODE model in [40] which shows that parameter values below the white curve yield a reversed cellular pH gradient and above the curve otherwise. The base case parameter set (see Table 1) is depicted in the figure by a “diamond”. In (A) 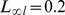, in (B) 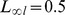. The grey shaed region in the inserts denote regions where a reversed pH gradient only occurs in the non-glycolytic cells (i.e. 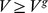).

Fig. 6 shows that the precence of a negative cellular pH gradient is strongly dependent on both the MCT activity, regulated by the parameter 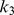, and the background production of intracellular H^+^-ions, 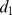, but also on the concentration of tissue lactate (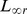). Not only is the negative cellular pH gradient attainable for a larger range of 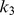 and 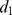 values when blood lactate level (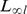) is lowered (compare A with B in Fig. 6), but, when tissue lactate 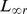 is low, a reversed cellular pH gradient occurs for the base case parameter values (as shown in Table 1, and described in detail in [40]) as indicated by a “diamond” in the figure. We take these base parameters as our most realistic set of values and so it is encouraging for parameter validation purposes that we can reproduce the much observed reversed cellular pH gradient with this parameter set. Note that we also highlight the region in this 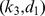-parameter space in which a reversed cellular pH gradient is predicted in a non-spatial well-mixed version of this model (below the white curves in Fig. 6, see [40] for full details). The key point here is that, for realistic parameters (including biologically reasonable variations to these values), we are unable to predict a reversed cellular pH gradient in the well mixed model for our base set of parameters. However, we can within this more realistic new spatial framework when tissue lactate is taken to be sufficiently low. Note also that there is a very small region of 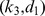-parameter space where a reversed cellular pH gradient emerges only for non-glycolytic cells. This occurs in the grey shaded region highlighted in the inserts of Fig. 6.

To interpret the above observations, we note that decreasing 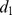 implies that the amount of H^+^-ions produced intracellularly is reduced, thereby favouring a more alkaline pH*_i_*. Increasing 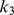 then increases the rate of removal of intracellular H^+^-ions to the extracellular space, which further reduces the pH*_i_* and increases pH*_e_* relative to pH*_i_* and therefore a negative cellular pH gradient emerges.

#### Varying the activity of NHEs and other sources of intracellular H^+^-ions

In contrast, we find that no matter how much the rate of activity of the NHEs, regulated by the parameter 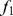, is varied the cellular pH gradient is reversed throughout the entire tissue section (for all values of 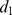, as long as MCT activity, 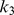, is sufficiently high). Straightforward calculation shows that 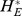 at steady state does not depend on 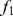 because the rate at which H^+^-ions enter the extracellular space, in our model, via the membrane-based transporters is equal to the rate of their removal by the blood vessel or their subsequent leakage into the cells. So, an increase in 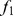 does not affect 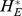 but will decrease 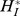 via extrusion by the NHE so that 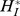 quickly becomes smaller than 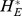 and the cellular pH gradient becomes reversed. In contrast, for higher values of tissue and blood lactate, the NHE only gives rise to a reversed cellular pH gradient provided that the level of other sources of H^+^-ions is small (i.e. 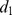 below a certain threshold). This is because increasing the levels of blood lactate means that extracellular lactate leaks into the blood at a lower rate. This then lowers the activity of the MCT (which functions according to the cellular lactate and H^+^ gradient) and consequently results in less intracellular H^+^-ions being transported outside the cell along with lactate.

Note that, if we artificially set intracellular and extracellular lactate to be equal, we find no cellular pH gradient reversal for the range of parameters that we have explored thus far. Crucially, this suggests that lactate plays a pivotal role in determining a reversed cellular pH gradient, which further motivates its inclusion as separate intracellular and extracellular components in the model.

To summarise, we have shown so far in this study that by allowing diffusion of the extracellular metabolites across the tissue section, we are able to recover a more realistic acidic negative cellular pH gradient throughout the tissue section–in the aerobic region as well as in the anaerobic region–which the well-mixed ODE model in [40] does not capture.

Our focus in the remainder of this study will be on the impact of a heterogeneous spatial distribution of NHEs and MCTs on the extent of the spatial cellular pH gradient reversal and on the spatial correlation of extracellular lactate and low pH*_e_*.

### Heterogeneous Distribution of MCT and NHE

**Figure 7:**
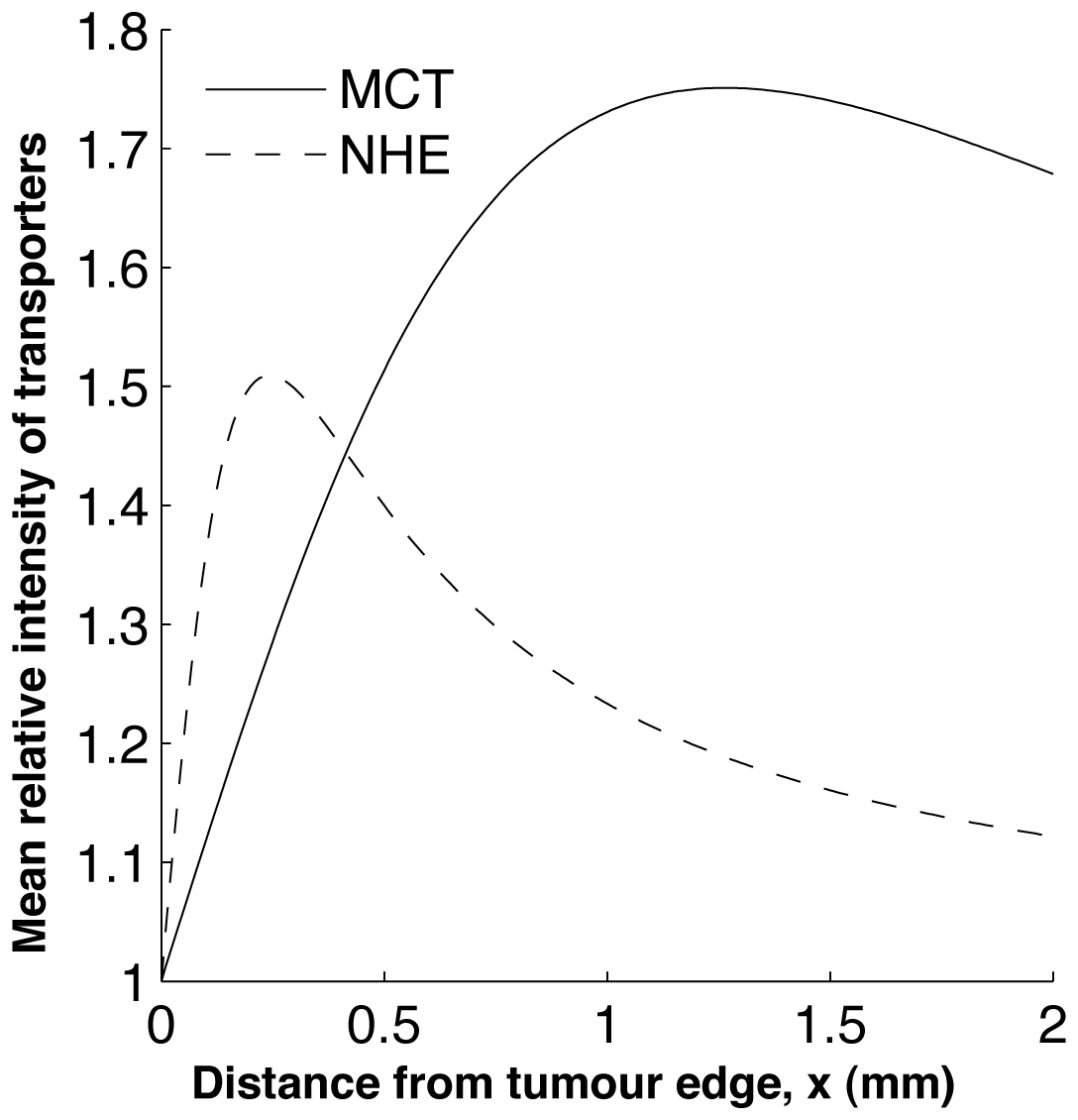
Spatial organisation of the NHE and the MCT in brain glioma as observed in [42]. Note that the average intensity of the transporters outside the tumour is set to one.

**Figure 8:**
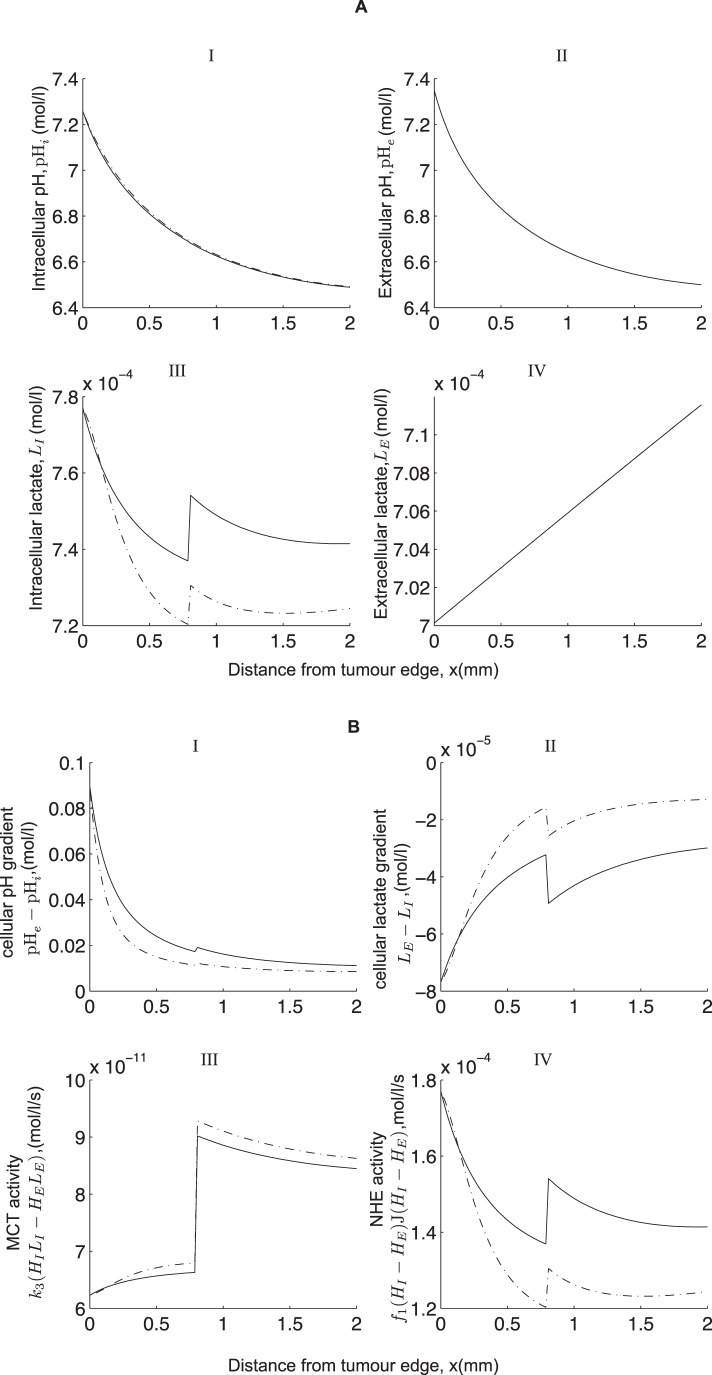
Effect of incorporating a heterogenous intensity of the NHE and MCT in the model. (A) shows the steady state profile of pH and lactate with heterogeneous (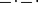) and homogeneous (–) functions representing the activity of the NHEs and MCTs. (B) Shows hydrogen and lactate gradients and the rates of activity of NHE and MCT. Parameter values are the same as in Fig. 3 but with 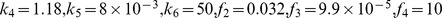 representing the heterogenous distribution of NHE and MCT. 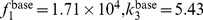.

So far, we have assumed that the MCTs and NHEs are homogeneously distributed on cells throughout the spatial domain. However, recent experiments carried out by Grillon *et al.* [42] on C6 rat gliomas reveal that the relative intensity of NHE peaks at a distance of 0.33±0.027 mm away from the tumour edge and that the intensity of MCT is also up-regulated at 1.05±0.14 mm from the edge of the tumour. The authors represent this spatial organisation of the transporters across the tumour rim graphically as shown in Fig. 7. With this in mind, we incorporate the following functions for the heterogenous expression of the NHE (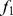) and the MCT (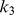), namely.
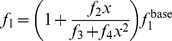


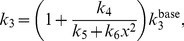
(9)where, 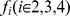, 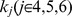 are chosen to best fit the curves shown in Fig. 7, and 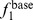, 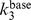 denote the base parameter values as presented in Table 1. It turns out, however, that incorporating these functions in our model has no qualitative effect on the pH and lactate profiles for the parameter values considered (see Fig. 8). Quantitatively, there is relatively little change to the levels of pH*_i_* and no observable change to pH*_e_* and 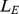. However, there is a noticeable change in the levels of intracellular lactate which is due to the variations in MCT activity. That is, intracellular lactate levels increase near the tumour edge due to a higher NHE activity there (compared to MCT activity) which leaves less intracellular H^+^ to bind with lactate for extrusion via the MCT and so intracellular lactate builds up. But as the NHE activity then drops further into the tumour (see Fig. 7), intracellular lactate begins to fall due to an increased MCT activity. However, this change in the NHE activity only appears to cause a small change in intracellular pH (see Fig. 8B(IV)). Therefore, it appears that the variations in MCT activity is the dominating factor here.

**Figure 9:**
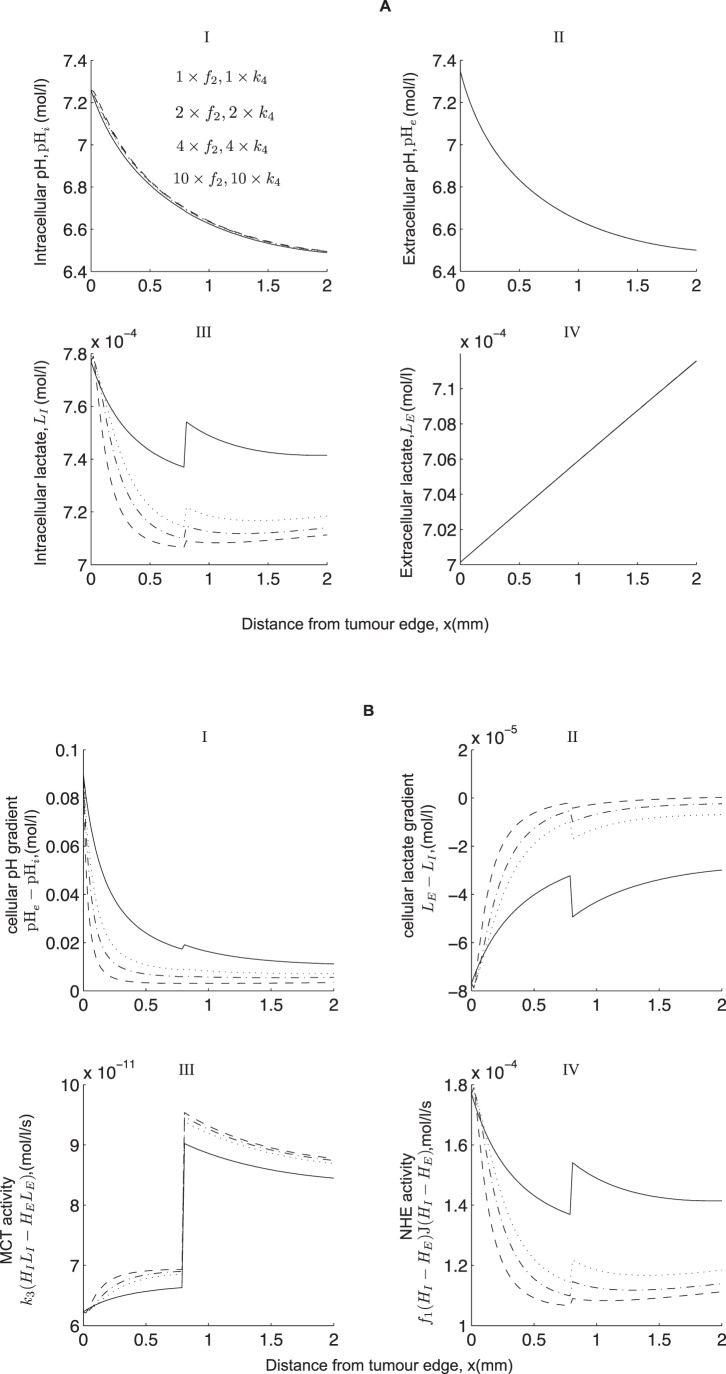
Effect of increasing the magnitude of the maximum rate of activity near the tumour rim of the NHE, 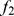, and MCT, 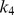. It appears that intracellular lactate is the most sensitive metabolite to variations in 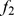 and 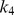. Intracellular pH is also affected, but to a lesser extent. (B) I shows pH gradient, (B) II lactate gradient, (B) III the MCT activity and (B) IV the NHE activity. Base values: 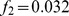, 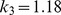. Remaining parameter values are the same as in the caption to Fig. 3.

We show in Fig. 9 that variations in the parameters that represent the maximum rate of activity of the NHE transporter, 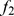, and the MCT transporter, 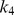, results in a qualitatively similar profile to that seen in Fig. 8. We see, however, a slight change in the qualitative profiles of intracellular lactate and intracellular pH near the blood vessel (near 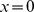). This is attributed to a sharp increase in the NHE activity and a slight dip in the MCT activity which results in a sharp increase in intracellular lactate levels there (see Fig. 9, III and IV). So in conclusion, our model suggests that an increased expression of the MCT and NHE near the tumour edge can affect the intracellular levels of lactate (the key effects being dominated by the MCT), but the effect on intracellular pH is much lower.

### The Inclusion of Intercellular Gap Junctions for H^+^-ions

It has been suggested that a lack of spatial correlation between an increase in extracellular lactate and a decrease in extracellular pH exists in some tumours because protons, which are exported extracellularly along with lactate in hypoxic regions, re-enter the cells indirectly via the 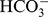/Cl^−^ exchanger or simply leak back into the cell and then are transported cell-to-cell via gap junctions to make protons available for the NHE exchanger [41]. In this section, we examine whether incorporating H^+^-ion intercellular gap junctions into our model has any effect on whether high extracellular lactate is correlated with a decrease in extracellular pH.

**Figure 10:**
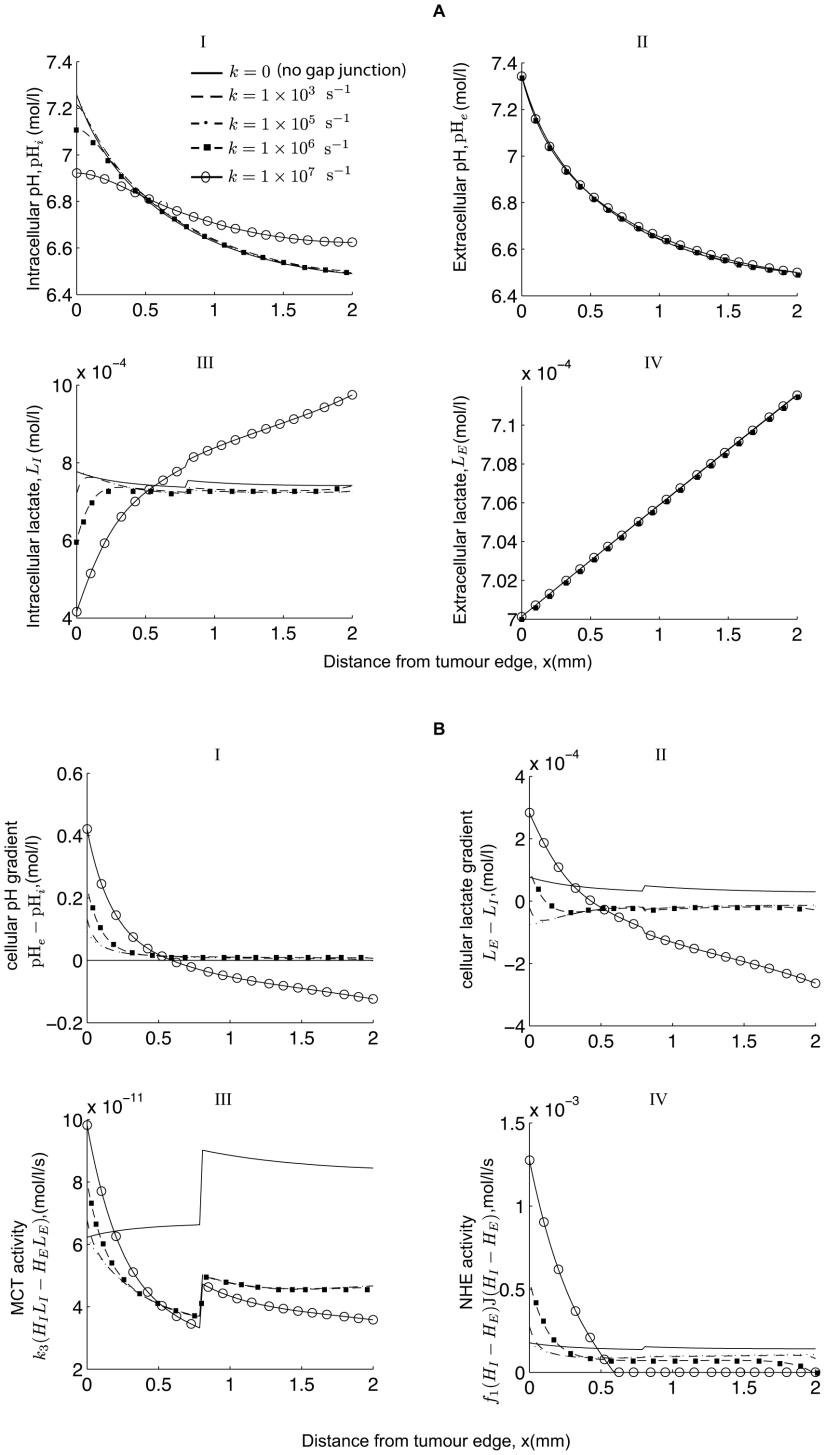
Effect of varying the rate of H^+^ transfer between cells, 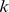, on pH and lactate profiles. Plots showing how the spatial profile of: (A) I 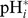 and (A) III 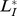 are affected as the rate of H^+^ transfer between cells, 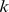, is varied and how little (A) II 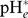 and (A) IV 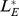 profiles change in comparison. (B) I shows pH gradient, (B) II lactate gradient, (B) III the MCT activity and (B) IV the NHE activity. Parameter values are the same as in the caption to Fig. 3.

We now introduce the following gap junction intercellular communication term,
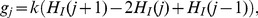
(10)into the spatially discretised form of equation (1), where 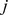 denotes the cell at spatial position 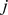, 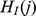 is the intracellular H^+^ -ion concentration in that cell and 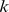 represents the rate of gap junction transfer of H^+^ between cells. Due to the lack of available data, we vary the magnitude of 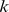 and illustrate the effect on the spatial profile of pH and lactate in Fig. 10.

We observe that as 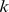 increases, the intracellular pH decreases in the aerobic region (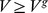) and increases in the glycolytic region (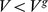) due to the intracellular H^+^-ions being transferred via gap junctions from the glycolytic region (where they are produced in excess) to the aerobic region. As a result, extracellular pH increases in the 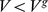 region (due to there being less intracellular H^+^-ions to be pumped outside the cell via the NHE or MCT) and decreases in the 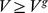 region (due to there being more intracellular H^+^-ions being pumped outside the cell). Extracellular lactate, however, remains largely insensitive and is essentially constant and this is attributed to its leakage into the surrounding tissue. However, we do see a slight increase in extracelluar lactate as the distance into the tissue section increases (see A IV) and note that the concentrations of extracellular H^+^ and lactate then become spatially correlated (i.e. high 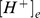 (low pH*_e_*) corresponding to high L*_E_*, see (A) II with (A) IV in Fig. 10). Admittedly, this is only a very weak correlation but is contrary to the hypothesis provided in [41], [42] which associates the activity of the H^+^ intercellular gap junctions with the lack of correlation between low pH*_e_* and high extracellular lactate. Also, intracellular lactate decreases in the 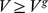 region (due to there being more intracellular H^+^-ions being pumped outside the cell along with lactate via the MCTs) and increases in the 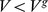 region (due to there being less intracellular H^+^-ions pumped outside the cell via the MCTs).

Furthermore, a reversed cellular pH gradient is observed in the glycolytic region, which is due to the intracellular H^+^-ions being transferred via the gap junctions from the glycolytic region (where they are produced in excess) to the aerobic region and thus lowering the levels of intracellular H^+^-ions in the glycolytic region and raising it in the aerobic region. Note that a sufficiently high value of gap junction transfer is required, 400 times quicker than the MCT and 10^6^ times quicker than the NHE activity, to see a significant effect on the cellular metabolite gradients (see Fig. 10).

## Discussion and Conclusions

One of the key questions that we wanted to answer in this work is can a spatial extension of our pH regulation model in [40] facilitate reversed cellular pH gradients such as that observed in many human tumours. The model in [40] is based on a well mixed framework and only predicts reversed cellular pH gradients for unrealistic pH values. We show in this article, however, that a simple spatial extension of this model, with no additional transporter or buffering terms, can predict reversed cellular pH gradients for much more realistic pH values, suggesting a potential critical role for the Na^+^/H^+^ and lactate/H^+^ transporters in the maintenance of such features. The second key question is whether lactate and H^+^-ions are spatially correlated. In other words, are their concentrations qualitatively similar throughout the tumour? One may intuitively assume that since lactate and H^+^ are produced together via glycolysis then the extracellular concentrations may then marry and, indeed, the diagnostic significance of high lactate has been implicated in numerous studies showing an association between high lactate and the incidence of metastasis [28], [29]. However, a recent study by Parkins et al [8] show that, under conditions of severe cell stress, the pH*_e_* declines in the absence of a corresponding accumulation of extracellular lactate and we indeed confirm such features in our model, highlighting that lactate should not be used as a standard indicator for extracellular acidity in tumours.

We have demonstrated in this spatial model that areas with high extracellular lactate can coincide with high extracellular H^+^-ion concentrations. However, when the rate of removal of H^+^-ions and lactate by blood vessels is reduced, lower extracellular lactate concentrations can exist where extracellular H^+^-ion concentrations are at their highest level. This result suggests a role for blood vessel perfusion rates in determining the spatial correlation of extracellular pH and lactate. Tumour blood vessels are chaotic and an order of magnitude leakier than normal vessels [68]. This is known to result in an increased interstitial fluid pressure inside tumours which can hamper the uptake of therapeutic agents [10]. Recently, Martin *et al.* [69] extended the acid-mediated tumour invasion model [36] by including the effect of vessel permeability on the acid gradient from the centre of the tumour to the normal tissue. They find that leaky vasculature (those with high vessel permeability) can lead to an overall acidification of the normal tissue further from the tumour boundary, and our present study agrees with this result (that is, if we take non-glycolytic cells to be representatives of normal cells).

The motivation of our work is to also determine the relative importance and inter-relationships between some of the main parameters involved in the spatial reversed cellular pH gradient, concentrating in particular on the influence of changes in tissue and blood lactate levels, background production of H^+^-ions and the activity of the MCTs and NHEs. We find that simply taking lower values of blood lactate levels gives a reversed cellular pH gradient throughout the spatial domain independent of the levels of tissue lactate. This is because, with lower blood lactate values, extracellular lactate leaks into the blood vessel until the level of lactate in the tissue equates that in the blood vessel. Hence, the lower the blood lactate level is, the lower the tissue extracellular lactate becomes, which in turn drives the MCTs to export lactate and H^+^-ions outside the cell at a higher rate due to the larger lactate gradient. This suggests that low levels of lactate found in the blood stream may indirectly cause a reversed cellular pH gradient in conjunction with an up-regulated tumour activity of the MCT. Likewise, we have found the existence of a reversed cellular pH gradient to be strongly dependent on the combined activity of the MCTs and background sources of H^+^-ion. In addition, we have found the cellular pH gradient to be always reversed no matter how high or low the NHE activity is, provided that the level of background sources of H^+^-ions is below a certain threshold. The ability of extracellular H^+^-ions to leak into the neighbouring blood vessel and to diffuse across the tissue from higher to lower concentrations means that the reversed cellular pH gradient occurs at more realistic values and is less alkaline (pH
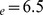
–7.4) than that observed in the well-mixed system we studied in [40] (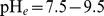). In terms of the significance of our results to anti-cancer therapy, we propose that decreasing intracellular H^+^ production rate and the activity of the MCT would lead to a normal cellular pH gradient and potentially aid in the uptake of some chemotherapeutic drugs.

Recent experiments carried out by Grillon *et al.* [42] on C6 rat gliomas reveal that the relative intensity of NHEs peak at a distance of 0.33±0.027 mm away from the edge of the tumour and that the intensity of MCTs is also up-regulated at 1.05±0.14 mm from the edge. The inclusion of heterogeneous expressions of the NHEs and the MCTs as in [42] has no qualitative effect on the model behaviour, but a considerable increase in their rate of activity can have a slight change on intracellular levels of lactate and intracellular pH. Quantitatively, this causes a significant difference to the intracellular levels of lactate which is attributed to the activity of the MCT. However, there is a relatively small effect on the intracellular pH. Based on our model, we therefore suggest that an up-regulated expression of NHE and MCT in the growing outer part of a tumour can give rise to a higher intracellular pH (which is known to aid tumour cell migration [70] and proliferation [71]) but may not result in a reversed cellular pH gradient or a redistribution of protons away from the glycolytic source. On the other hand, including intercellular gap junction communication can give rise to a reversed cellular pH gradient. Note that in order for this to happen, intercellular gap H^+^-ion transfer needs to be much more rapid compared to the other transporter processes in the model (i.e. 400 times quicker than the MCT and 10^6^ times quicker than the NHE activity). This results in intracellular H^+^-ions being transferred from the glycolytic region (where they are produced in excess) to the aerobic region and thus lowering the levels of intracellular H^+^-ions in the glycolytic region and raising it in the aerobic region.
