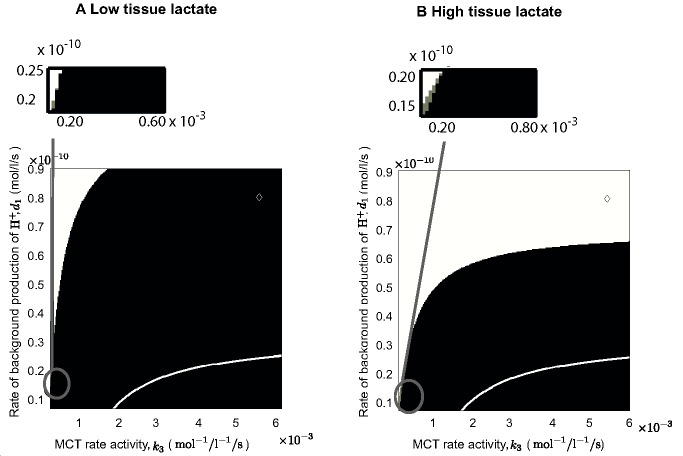# Correction: Theoretical Predictions of Lactate and Hydrogen Ion Distributions in Tumours

**DOI:** 10.1371/annotation/d5a0e265-b707-476d-9975-3278f9defabf

**Published:** 2013-10-30

**Authors:** Maymona Al-Husari, Steven D. Webb

There were errors in Figure 6. The correct version of the figure is available here: 

**Figure:**